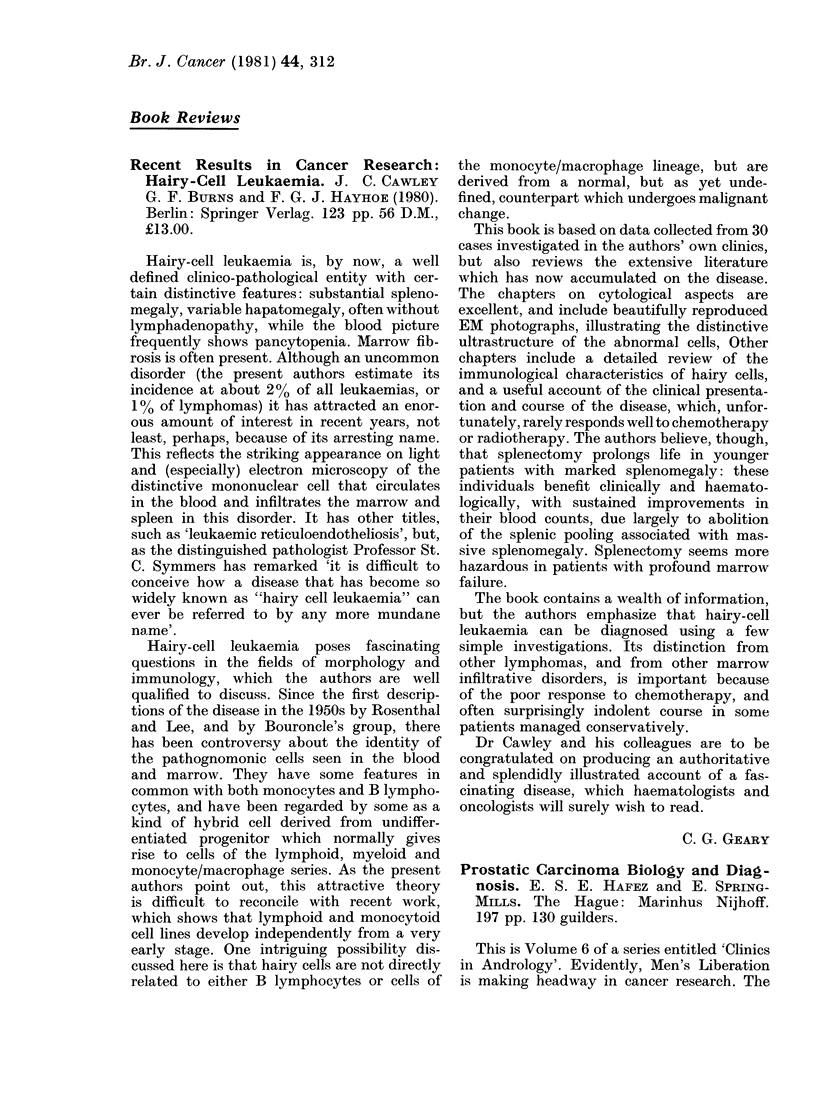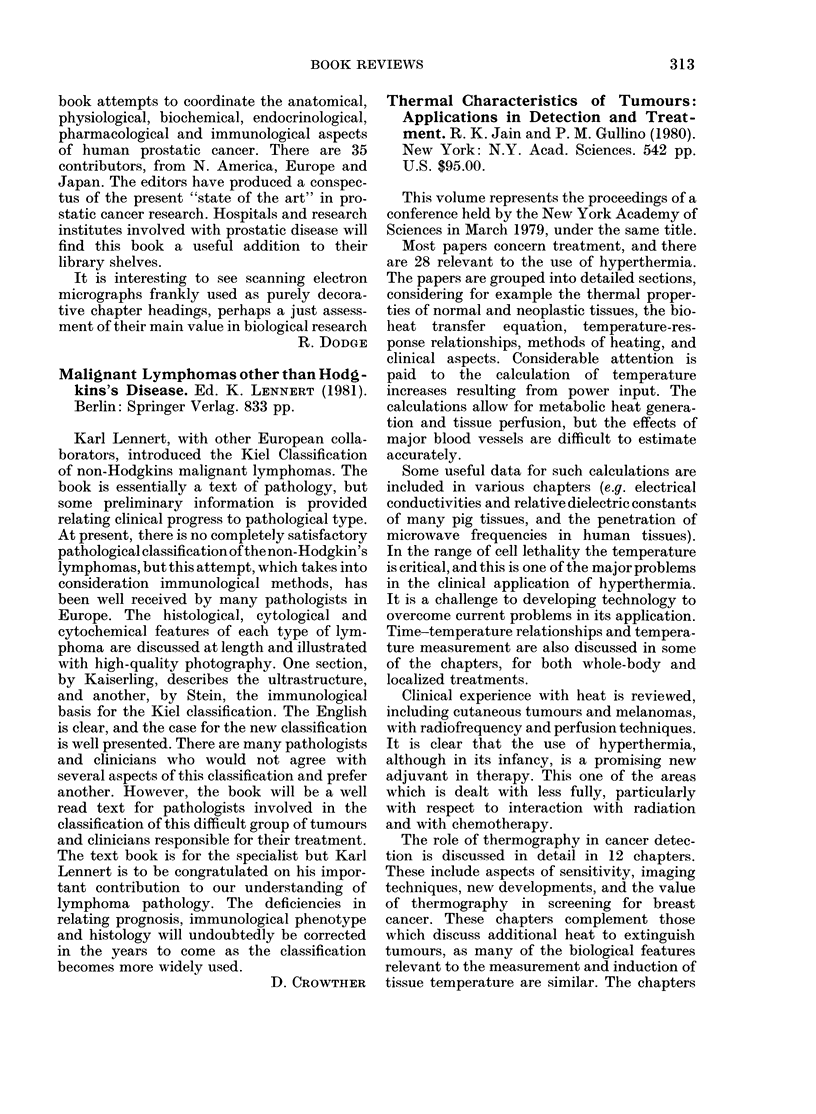# Prostatic Carcinoma Biology and Diagnosis

**Published:** 1981-08

**Authors:** R. Dodge


**Prostatic Carcinoma Biology and Diagnosis.** E. S. E. HAFEZ and E. SPRING-MILLS. The Hague: Marinhus Nijhoff. 197 pp. 130 guilders.

This is Volume 6 of a series entitled 'Clinics in Andrology'. Evidently, Men's Liberation is making headway in cancer research. The book attempts to coordinate the anatomical, physiological, biochemical, endocrinological, pharmacological and immunological aspects of human prostatic cancer. There are 35 contributors, from N. America, Europe and Japan. The editors have produced a conspectus of the present "state of the art" in prostatic cancer research. Hospitals and research institutes involved with prostatic disease will find this book a useful addition to their library shelves.

It is interesting to see scanning electron micrographs frankly used as purely decorative chapter headings, perhaps a just assessment of their main value in biological research

R. Dodge